# SirT1 modulates the estrogen–insulin-like growth factor-1 signaling for postnatal development of mammary gland in mice

**DOI:** 10.1186/bcr1632

**Published:** 2007-01-03

**Authors:** Hongzhe Li, Grace K Rajendran, Ninning Liu, Carol Ware, Brian P Rubin, Yansong Gu

**Affiliations:** 1 Department of Radiation Oncology, University of Washington School of Medicine, Seattle, WA 98195, USA; 2 Department of Immunology, University of Washington School of Medicine, Seattle, WA 98195, USA; 3 Department of Comparative Medicine, University of Washington School of Medicine, Seattle, WA 98195, USA; 4 Department of Pathology, University of Washington School of Medicine, Seattle, WA 98195, USA; 5 Department of Molecular Genetics, Lerner Research Institute, Cleveland, OH 44195, USA

## Abstract

**Introduction:**

Estrogen and insulin-like growth factor-1 (IGF-1) play important roles in mammary gland development and breast cancer. SirT1 is a highly conserved protein deacetylase that can regulate the insulin/IGF-1 signaling in lower organisms, as well as a growing number of transcription factors, including NF-κB, in mammalian cells. Whether SirT1 regulates the IGF-1 signaling for mammary gland development and function, however, is not clear. In the present study, this role of SirT1 was examined by studying SirT1-deficient mice.

**Methods:**

SirT1-deficient (SirT1^ko/ko^) mice were generated by crossing a new strain of mice harboring a conditional targeted mutation in the SirT1 gene (SirT1^co/co^) with CMV-Cre transgenic mice. Whole mount and histology analyses, immunofluorescence staining, immunohistochemistry, and western blotting were used to characterize mammary gland development in virgin and pregnant mice. The effect of exogenous estrogen was also examined by subcutaneous implantation of a slow-releasing pellet in the subscapular region.

**Results:**

Both male and female SirT1^ko/ko ^mice can be fertile despite the growth retardation phenotype. Virgin SirT1^ko/ko ^mice displayed impeded ductal morphogenesis, whereas pregnant SirT1^ko/ko ^mice manifested lactation failure due to an underdeveloped lobuloalveolar network. Estrogen implantation was sufficient to rescue ductal morphogenesis. Exogenous estrogen reversed the increased basal level of IGF-1 binding protein-1 expression in SirT1^ko/ko ^mammary tissues, but not that of IκBα expression, suggesting that increased levels of estrogen enhanced the production of local IGF-1 and rescued ductal morphogenesis. Additionally, TNFα treatment enhanced the level of the newly synthesized IκBα in SirT1^ko/ko ^cells. SirT1 deficiency therefore affects the cellular response to multiple extrinsic signals.

**Conclusion:**

SirT1 modulates the IGF-1 signaling critical for both growth regulation and mammary gland development in mice. SirT1 deficiency deregulates the expression of IGF-1 binding protein-1 and attenuates the effect of IGF-1 signals, including estrogen-stimulated local IGF-1 signaling for the onset of ductal morphogenesis. These findings suggest that the enzymatic activity of SirT1 may influence both normal growth and malignant growth of mammary epithelial cells.

## Introduction

Mammalian SirT1 belongs to a family of nicotinamide adenine dinucleotide-dependent histone deacetylases [1,2]. SirT1 is most closely related to yeast Sir2, the founding member of the evolutionarily conserved Sir2 family. Yeast Sir2 is required for silencing transcription at the telomeric region and mating type loci, and for suppression of ribosomal DNA recombination [3,4]. The expression of an extra copy of Sir2 in either yeast mother cells or multicell organisms such as nematodes can significantly extend the lifespan [5,6]. Inactivation of Sir2 enhances stress resistance and extends chronological lifespan of nondividing yeast cells, which is opposite to the requirement for Sir2 function in the reproductive lifespan [7]. Whether SirT1 regulates the reproductive lifespan and/or the chronological lifespan in mammals remains unknown.

Sir2 is an integral part of an evolutionarily conserved insulin/insulin-like growth factor-1 (IGF-1) signaling (IIS) system in worms (*Caenorhabditis elegans*), fruit flies (*Drosophila*), mice, and humans [8,9]. The IIS system includes membrane-bound receptors, cytoplasmic kinases, and nuclear transcription factors. To maintain the proper expression of the effector genes for the IIS system, these conserved components form a sophisticated regulatory system, which centers on a family of forkhead transcription factors (forkhead box 'other' proteins (FoxOs)), and operates on two levels. On one level, SirT1-mediated protein deacetylation attenuates the transcriptional activity of nuclear FoxO transcription factors [10-12]. On the second level, the FoxO transcription factors can be sequestered within the cytoplasm when phosphorylated by activated Akt kinases in response to insulin and IGF-1 signals [13]. Conceivably, the IIS system senses the levels of insulin and IGF-1 and negatively regulates the expression of the effector genes. The IIS system is responsible for food storage, stress tolerance, and longevity in lower organisms, such as *C. elegans *[8,9,14]. In more advanced species, steroid hormones evolved to regulate the IIS system [15]. In mice and humans, the IGF-1 signaling of the IIS system mediates local effects for growth and hormonal regulation for multiple tissues, including mammary glands [16,17].

Mammalian SirT1 has evolved to modify the activity of a growing number of transcription factors, including p53, NF-κB, and PGC-1α, suggesting that SirT1 functions in a wide range of cellular responses to stress, inflammation, and nutrients [18-21]. SirT1-deficient mice display characteristic phenotypes of perinatal death and growth retardation as well as other diverse phenotypes, such as eye defects, with varying severity [22,23]. The underlying causal mechanism for these phenotypes, however, remains unknown. We recently generated SirT1-deficient (SirT1^ko/ko^) mice and found that both male and female SirT1^ko/ko ^mice can be fertile, which is in contrast to the sterile phenotypes observed in one strain of SirT1-deficient mice [22]. This led to our study of the link between SirT1 and IGF-1 signaling using the mammary gland as a model organ.

The mammary gland is a unique organ because it develops after birth and undergoes dynamic changes throughout the reproductive lifespan of a female. At the onset of puberty, ovarian estrogen stimulates ductal morphogenesis during which mammary epithelial progenitor cells differentiate and proliferate while interacting with adipocytes and stromal cells within mammary fat pad [17,24,25]. Ovarian estrogen, in synergy with pituitary growth hormone (GH), stimulates stromal cells to produce local IGF-1. The local IGF-1, but not liver-produced systemic IGF-1, provides a paracrine signal for commencing ductal morphogenesis [26]. Mice lacking GH, estrogen, IGF-1, GH receptor, or estrogen receptor alpha (ERα) fail to undergo postnatal ductal morphogenesis [17,24-31], indicating that both steroid hormones and IGF-1 are on the common pathway for a critical developmental checkpoint. Once the arborated ductal network is established, cycles of differentiation, proliferation, and death of secretory alveolar epithelium repeat with each pregnancy [17,24,25].

In the present article we report the finding and characterization of impeded ductal morphogenesis in virgin SirT1^ko/ko ^mice and lactation failure in SirT1^ko/ko ^mothers. The characterization of these phenotypes has identified a SirT1-dependent regulatory mechanism by which SirT1 modulates the effectiveness of the estrogen–IGF-1 signaling for mammary gland development. The estrogen–IGF-1 signaling is defined as the ovarian estrogen-regulated, stromal cell-produced local IGF-1 signal for stimulating mammary epithelial cells.

## Materials and methods

### Mice

A previously described SirT1 targeting construct, KOII [23], was used to generate mice harboring a conditional targeted mutation in the SirT1 gene (SirT1^co/co ^mice) (see Additional file 1). The breeding of SirT1^co/co ^mice and CMV-Cre transgenic mice results in mice harboring a germline-transmitted deletion of exon 4 of the SirT1 gene (SirT1^+/ko ^mice). Both SirT1^co/co ^mice and SirT1^+/co ^mice were used to establish breeding colonies for generating SirT1^co/co ^and SirT1^ko/ko ^mice, respectively. Both SirT1^co/co ^mice and SirT1^ko/ko ^mice were in a mixed 129SvJ/C57B6 background. Mice were housed in a special-pathogen-free facility and all procedures were approved by the University of Washington Animal Care and Use Committee. A PCR-based genotyping method was established to identify the wild-type, co, and ko loci of the SirT1 gene using three primers: 5' co primer, 5'-GGTTGACTTAGGTCTTGTCTG; 5' ko primer, 5'-AGGCGGATTTCTGAGTTCGA; 3' primer, 5'-CGTCCCTTGTAATGTTTCCC. Murine embryonic fibroblasts (MEFs) were isolated from the embryos between embryonic day 12.5 and embryonic day 14.5. The body weight was measured once a week.

### Serum insulin-like growth factor-1

A commercial radioimmunoassay (ALPCO Inc., Windham, NH, USA) was used. Samples (300 μl) were prepared via an alcohol extraction followed by a 2-day disequilibrium incubation at 4°C. Samples were measured in duplicate and data were calculated as the samples were counted. Six standards ranging from 10 to 500 ng/ml were used to generate the standard curve. The lower limit of detection was 10 ng.

### Survival assay

To determine the sensitivity of embryonic stem cells after ionizing radiation or hydrogen peroxide treatment, 300 embryonic stem cells were seeded onto a 10-cm plate for 24 hours. The plates were either exposed to a ^137^Cs source at indicated doses or were treated with embryonic stem media containing hydrogen peroxide at indicated concentrations for 30 minutes. Seven days after irradiation or hydrogen peroxide treatment, the embryonic stem colonies stained with crystal violet were counted.

### Estrogen implantation

Estrogen pellets in the form of 17β-estradiol, at 0.1 mg, and the 21-day releasing time were obtained from Innovative Research (Sarasota, FL, USA). One pelletwas subcutaneously implanted in the subscapular region ofindividual female mice using a sterilizedmetal trochar (Innovative Research).

### Whole mount analysis

The inguinoabdominal mammary fat pads were spread on microscope slides, fixed in Carnoy's fixative overnight, hydrated and stained with carmine alum stain (Sigma, St. Louis, MO, USA) overnight, and were then dehydrated, treated with xylene to remove fat, and mounted with Secure Mount (Fisher Scientific, Pittsburgh, PA, USA) and cover slips.

### Histological, immunohistochemical, and immunofluorescence analyses

The mice were given a single dose of bromodeoxyurdine (BrdU) at 100 g/kg body weight via intraperitoneal injection 2 hours before euthanasia. The mammary fat pads, as well as other tissues, were collected and fixed in 10% formalin solution (Fisher Scientific). Paraffin-embedded sections were prepared at 4 μm thickness followed by standard H & E staining for histological analysis and by immunohistochemical staining for BrdU-labeled cells (Sigma) and apoptotic cells (Promega, Madison, WI, USA). To detect the presence of mouse milk proteins, a rabbit anti-mouse milk specific protein antibody (Nordic Immunology, Tillburg, The Netherlands) was used for immunofluorescent staining, followed by Texas Red anti-rabbit secondary antibody (Molecular Probes) and DAPI staining for nuclei (Molecular Probe, Eugene, OR, USA). Microscopic analyses of all histological findings were carried out on an AxioVert 200M microscope with AxioVision 4.5 software (Carl Zeiss, München-Hallbergmoos, Germany).

### Protein analysis

Protein extracts were prepared from mammary tissues as well as from MEFs. The following primary antibodies were used: anti-SirT1 (Upstate Biotechology, Lake Placid, NY, USA), anti-IGF-1 binding protein-1 (IGFBP-1) (R&D Systems, Minneapolis, MN, USA), and anti-actin and anti-IκBα (Santa Cruz Biotechnology, Santa Cruz, CA, USA). The corresponding secondary antibodies were horseradish peroxidase-conjugated anti-rabbit (Pierce, Rockford, IL, USA), anti-rat, and anti-goat (Santa Cruz Biotechnology).

## Results

### Growth retardation in SirT1^ko/ko ^mice

**Figure 1:**
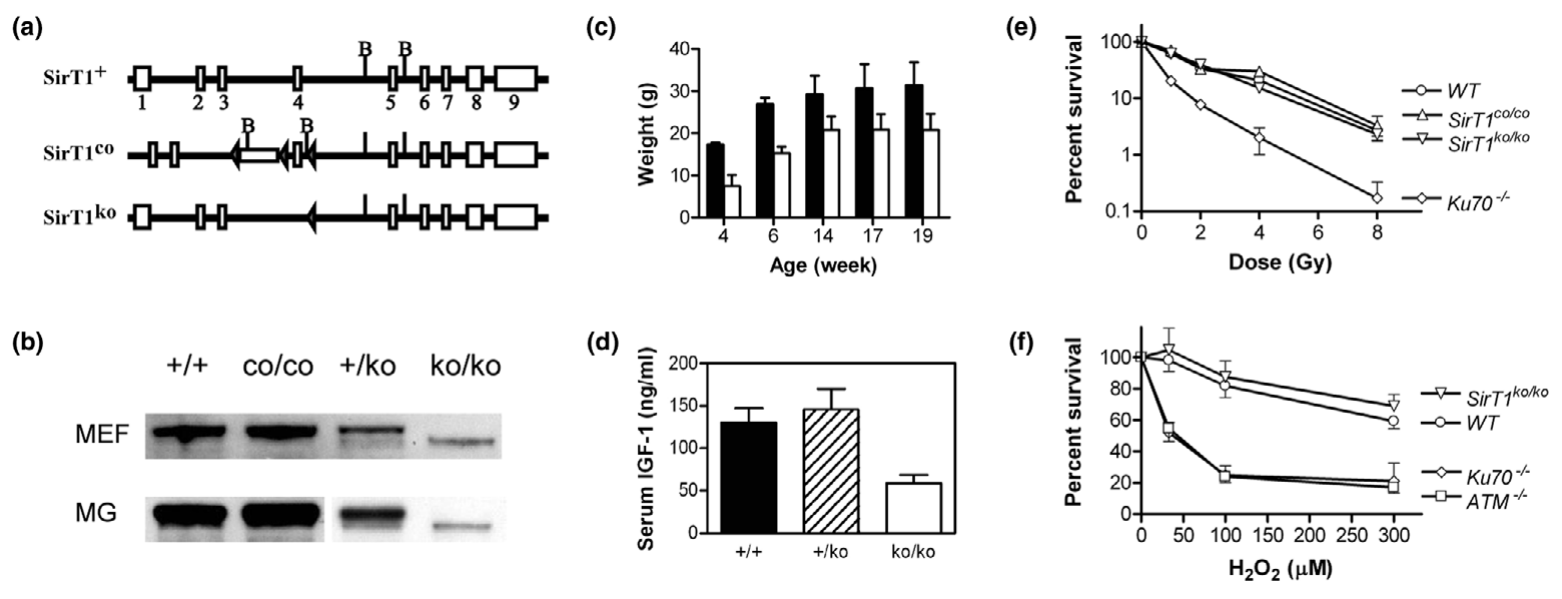
**Generation and characterization of SirT1^co/co ^and SirT1^ko/ko ^mice**. **(a) **Mouse SirT1 wild-type allele (SirT1^+^), conditional targeted allele (SirT1^co^), and knockout allele (SirT1^ko^). B, *BamH*I. **(b) **Western blot analysis of SirT1 expression in wild-type (+/+), conditional targeted (co/co), heterozygote (+/ko), and knockout (ko/ko) murine embryonic fibroblasts (MEFs) and mammary tissues (MG). **(c) **Growth retardation in surviving SirT1^ko/ko ^mice (open bar) using sibling mice or age-matched mice of control genotypes (filled bar). **(d) **The serum levels of insulin-like growth factor-1 (IGF-1) in wild-type (+/+), SirT1^+/ko ^(+/ko), and SirT1^ko/ko ^(ko/ko) mice. **(e) **The survival curves of wild-type (WT), SirT1^co/co^, SirT1^ko/ko^, and Ku70^-/- ^embryonic stem cells after ionizing radiation. **(f) **The survival curve of wild-type (WT), SirT1^ko/ko^, Ku70^-/-^, and Atm^-/- ^embryonic stem cells after treatment with hydrogen peroxide.

The conditional targeted SirT1 mutant mice (SirT1^co/co ^mice) carry an insertion mutation of the neomycin-resistant gene and lox sequences in the SirT1 gene flanking exon 4 that encodes a conserved Sir2 motif (Figure 1a). The mutation does not affect the expression of SirT1 in SirT1^co/co ^mice (Figure 1b). As expected, SirT1^co/co ^mice are phenotypically indistinguishable from wild-type mice. To convert the SirT1 co allele into the SirT1 ko allele, SirT1^co/co ^mice were crossed with CMV-Cre transgenic mice to generate SirT1 heterozygotes carrying the SirT1^+/ko^, CMV-Cre^+ ^genotype. The expression of the CMV-Cre transgene catalyzes the deletion of exon 4 in most lineages of cells, including germ cells (Figure 1a). This SirT1 ko allele should be identical to the previously described Δex4 mutation [23]. SirT1^+/ko^, Cre^+ ^mice were backcrossed with wild-type mice to generate the mice harboring a germline-transmitted deletion mutation (that is, SirT1^+/ko ^mice). The breeding of SirT1^+/ko ^male mice and SirT1^+/ko ^female mice resulted in SirT1^ko/ko ^mice. The cells derived from either SirT1^+/ko ^mice or SirT1^ko/ko ^mice expressed a SirT1 mutant protein due to the inframe deletion of exon 4 (Figure 1b). This SirT1 mutant protein may not be functional, however, since SirT1^+/ko ^mice are phenotypically indistinguishable from wild-type mice and SirT1Δ^ex4/Δex4 ^mice were phenotypically identical to SirT1 null mice [23].

Similar to other strains of SirT1-deficient mice, nearly two-thirds of SirT1^ko/ko ^newborns die shortly after birth and the majority of surviving SirT1^ko/ko ^mice manifest growth retardation (Table 1 and Figure 1c) [22,23]. Growth retardation may result from a systemic defect in hormonal regulation, DNA double-strand break repair, or other causal mechanisms. We found that SirT1^ko/ko ^mice have reduced levels of serum IGF-1 (Figure 1d). IGF-1 often acts as a local effector for pituitary GH. The serum level of GH in SirT1^ko/ko ^mice, however, appeared to be within the normal range (data not shown). On the other hand, yeast Sir2 and its associated Sir complex, as well as mouse SirT6, have also been implicated in DNA double-strand break repair and the maintenance of chromosome stability [32-34]. We found that SirT1^ko/ko ^embryonic stem cells do not have increased sensitivity to either ionizing radiation or hydrogen peroxide treatment when compared with wild-type cells, or with positive control cells such as Ku70-deficient or Atm-deficient cells (Figure 1e,f). Both Ku70-deficient mice and Atm-deficient mice display growth retardation phenotypes [35,36]. The growth retardation phenotype in SirT1^ko/ko ^mice is therefore probably not due to a defect in DNA damage repair, but rather results from a deficit in IGF-1 signaling.

### Lactation failure

Both male and female SirT1^ko/ko ^mice can be fertile, which is in contrast with the reported sterile phenotype in one strain of SirT1-deficient mice [22]. When male SirT1^ko/ko ^mice impregnated female SirT1^+/ko ^mice, the number of surviving SirT1^ko/ko ^offspring was reduced due to the partial perinatal lethal phenotype (Table 1). In a reciprocal approach, male SirT1^+/ko ^mice can also impregnate female SirT1^ko/ko ^mice (Table 1). After parturition, SirT1^ko/ko ^mothers exhibited normal nursing behavior. The pups did not survive for more than 3 days after birth, however, unless they were immediately removed and put under the care of a foster mother. All pups died of dehydration or starvation as a result of lack of milk in their stomachs.

**Figure 2:**
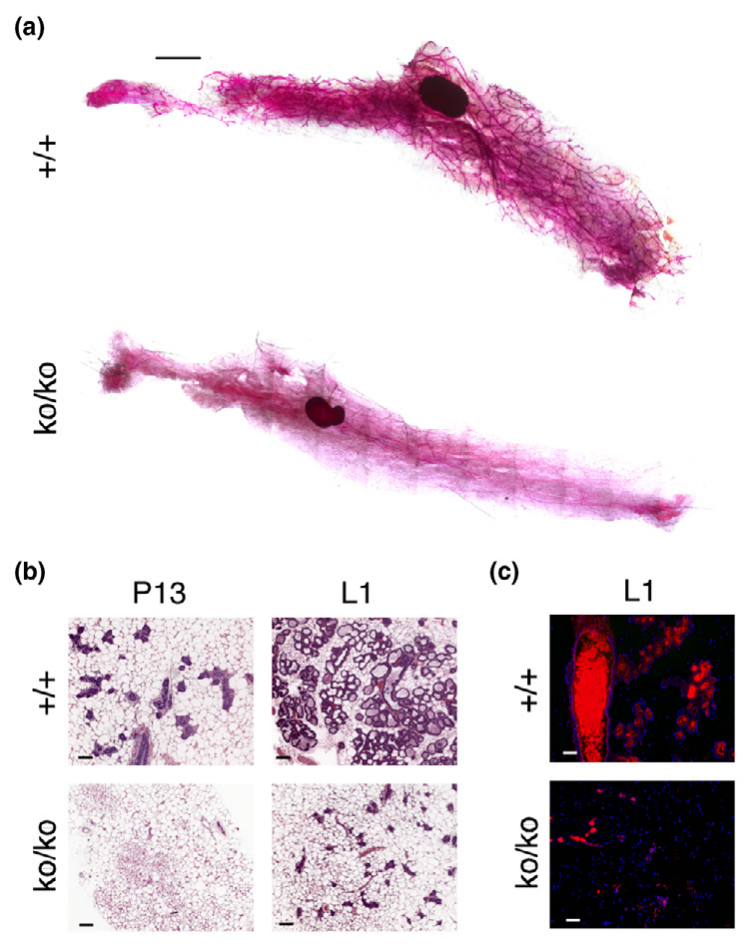
**Lactation failure in SirT1^ko/ko ^mice**. **(a) **Whole mount analysis of the mammary tissues from virgin wild-type (+/+) and SirT1^ko/ko ^mice (ko/ko). Scale bar = 2 mm. **(b) **Histological analysis of the mammary tissues harvested from wild-type (+/+) and SirT1^ko/ko ^female mice on either day 13 of pregnancy (P13) or lactation day 1 (L1). Scale bars = 100 μm. **(c) **Immunofluorescence staining of milk production in the mammary tissues from wild-type (+/+) and SirT1^ko/ko ^mice on L1. Red and smear stains, mouse milk proteins; blue dots, nuclear staining of adipocytes, epithelial cells, and other cells in mammary tissues. Scale bars = 100 μm.

To determine whether the SirT1^ko/ko ^mothers encountered a lactation defect, whole mount and histological analyses were used to characterize the morphological changes in the mammary glands. We found that virgin SirT1^ko/ko ^mice displayed impeded ductal morphogenesis up to 9 months of age, while age-matched virgin wild-type mice displayed extensive ductal elongation and branching (Figure 2a). The absence of ductal morphogenesis persisted in pregnant SirT1^ko/ko ^mice up to day 13 of pregnancy (Figure 2b). Despite the fact that pregnancy can induce ductal morphogenesis at the late stage of pregnancy, underdeveloped mammary glands in SirT1^ko/ko ^female mice cause a severe deficit in milk production, as shown by histological analysis as well as the immunofluorescence analysis using an anti-milk antibody (Figure 2b,c).

### Pregnancy-induced mammary gland development

**Figure 3:**
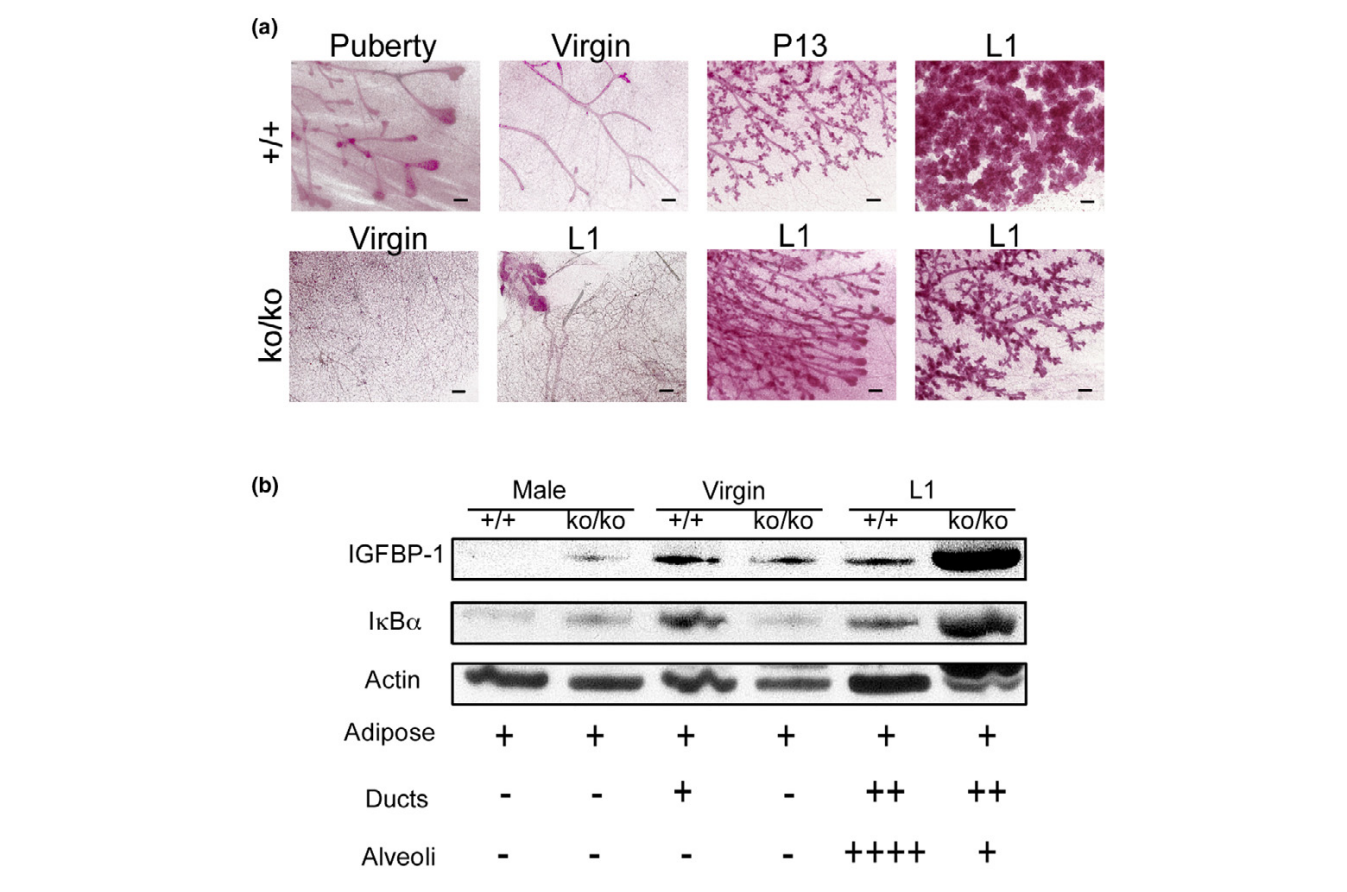
**Pregnancy-induced ductal morphogenesis**. **(a) **Whole mount analysis of mammary gland development. Upper panel: wild-type (+/+) mice show terminal end buds (TEBs) at the onset of puberty, elongated ducts (virgin mice), site branching during pregnancy (P13), and lobuloalveolar structures for milk production on lactation day 1 (L1). Lower panel: virgin SirT1^ko/ko ^(ko/ko) mice show impeded ductal morphogenesis. Pregnancy-induced ductal morphogenesis manifests transitional TEBs, ductal elongation, and side branching with variety in three SirT1^ko/ko ^mice on L1. All scale bars = 200 μm. **(b) **Western blot analysis of both insulin-like growth factor-1 binding protein-1 (IGFBP-1) and IκBα in mammary tissues of male mice, virgin mice, and L1 female mice of wild type (+/+) and SirT1^ko/ko ^(ko/ko). Actin is used as a loading control. Lower panel scores the estimated density of indicated lineages of cells in the protein extracts, which are based on the morphological analyses in (a).

To characterize the developmental defect leading to lactation failure, we analyzed the mammary glands of SirT1^ko/ko ^female mice on lactation day 1. Terminal end buds (TEBs) are club-shaped transitional structures (see Figure 3a, upper panel). In wild-type female mice, TEBs form and precede ductal elongation and branching at the onset of puberty [17,24,25]. When TEBs/ducts reach the edge of mammary fat pads, TEBs regress as shown in the virgin mice in Figure 3a (upper panel). Interestingly, the transitional TEBs can be readily identified in SirT1^ko/ko ^female mice on lactation day 1, manifesting as either newly formed TEBs or as TEBs attached to developing ductal and alveolar structures (Figure 3a, lower panel). Moreover, SirT1^ko/ko ^mice displayed varying degrees of ductal development, ranging from a lack of any ductal structure to a fully developed ductal network, the latter of which is comparable with the morphology and cellularity of wild-type mammary tissues on day 13 of pregnancy (Figure 3a). These observations indicated that pregnancy could rescue impeded ductal morphogenesis in virgin SirT1^ko/ko ^mice.

Normal alveolar morphogenesis takes place under the control of additional hormones and growth factors during pregnancy and lactation [17,24,25]. These coordinated efforts are necessary to support a densely saturated lobuloalveolar system for secreting milk after parturition (see wild-type mice on lactation day 1 in Figures 2b and 3a). In some SirT1^ko/ko ^mice, the alveolar morphogenesis was initiated, as indicated by the presence of a few secretory alveolar epithelial cells within scattered alveolar structures sprouted from a well-established ductal branching network (Figures 2c and 3a, lower panel). The extent of alveolar morphogenesis in SirT1^ko/ko ^mice was inadequate, however, as shown by the severe deficit in milk production when compared with that in wild-type mice (Figure 2c).

### Exogenous estrogen-induced ductal morphogenesis

**Figure 4:**
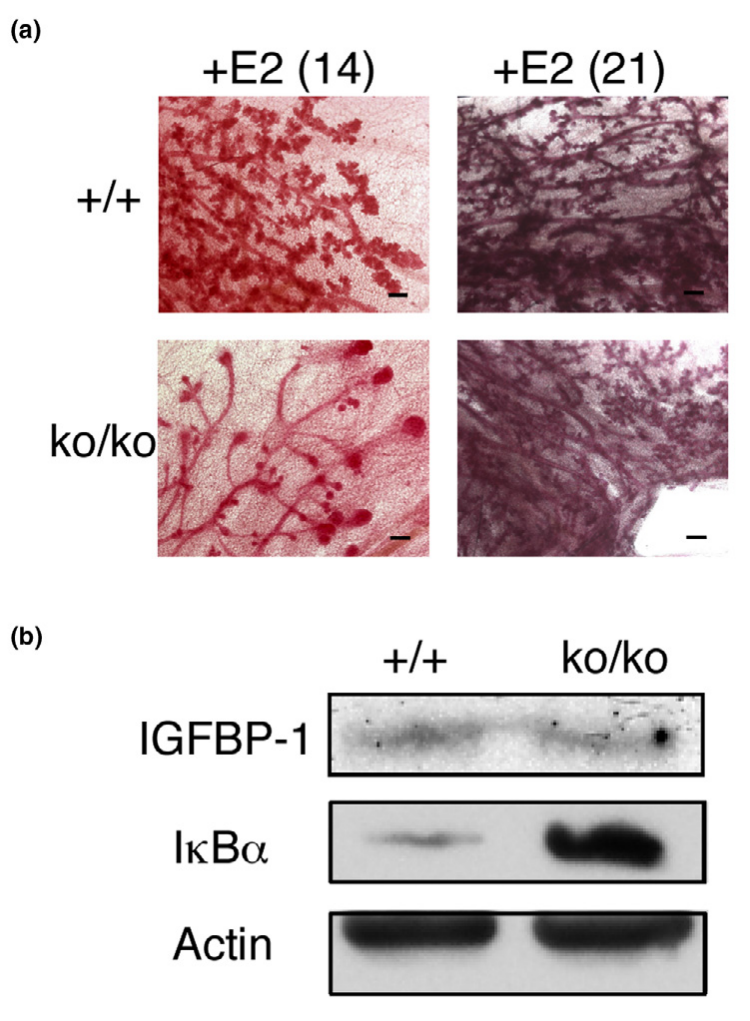
**Estrogen implantation stimulates ductal elongation and site branching**. **(a) **Whole mount analysis of the mammary tissues from the virgin wild-type (+/+) and SirT1^ko/ko ^mice implanted with estrogen pellets (+E2) on day 14 and day 21. Scale bars = 200 μm. **(b) **Western blot analysis of the expression of insulin-like growth factor-1 binding protein-1 (IGFBP-1) and IκBα, with actin as a loading control, in mammary tissues from the virgin wild-type (+/+) and SirT1^ko/ko ^mice implanted with estrogen pellets on day 21.

To test whether the increased levels of estrogen in pregnant mice were sufficient to induce ductal morphogenesis in SirT1^ko/ko ^mice, a single estrogen pellet was implanted in the subscapular region of each virgin SirT1^ko/ko ^mouse and the mammary tissues were analyzed 14 and 21 days after the implantation. By day 14, wild-type mice displayed ductal side branching reminiscent of the morphological changes during early pregnancy (Figure 4a, left panel). In contrast, SirT1^ko/ko ^mice showed TEBs and ductal elongation, which are the characteristic features of ductal morphogenesis in pubertal wild-type mice. By day 21, ductal elongation and side branching were restored in SirT1^ko/ko ^mice and the extent of ductal morphogenesis was indistinguishable between wild-type and SirT1^ko/ko ^female mice (Figure 4a, right panel). These observations clearly indicated that exogenous estrogen alone is sufficient to rescue ductal morphogenesis in virgin SirT1^ko/ko ^mice. Furthermore, the presence of transitional TEBs on day 14 and of ductal side branching on day 21 suggested that the characteristic features of mammary gland development at puberty and during early pregnancy could be coupled in response to increasing levels of estrogen. Increased levels of estrogen, either during pregnancy or through implantation, were therefore sufficient to stimulate the differentiation of epithelial progenitor cells and ductal morphogenesis in virgin SirT1^ko/ko ^mice.

### Concurrent mammary epithelial cell proliferation and differentiation

**Figure 5:**
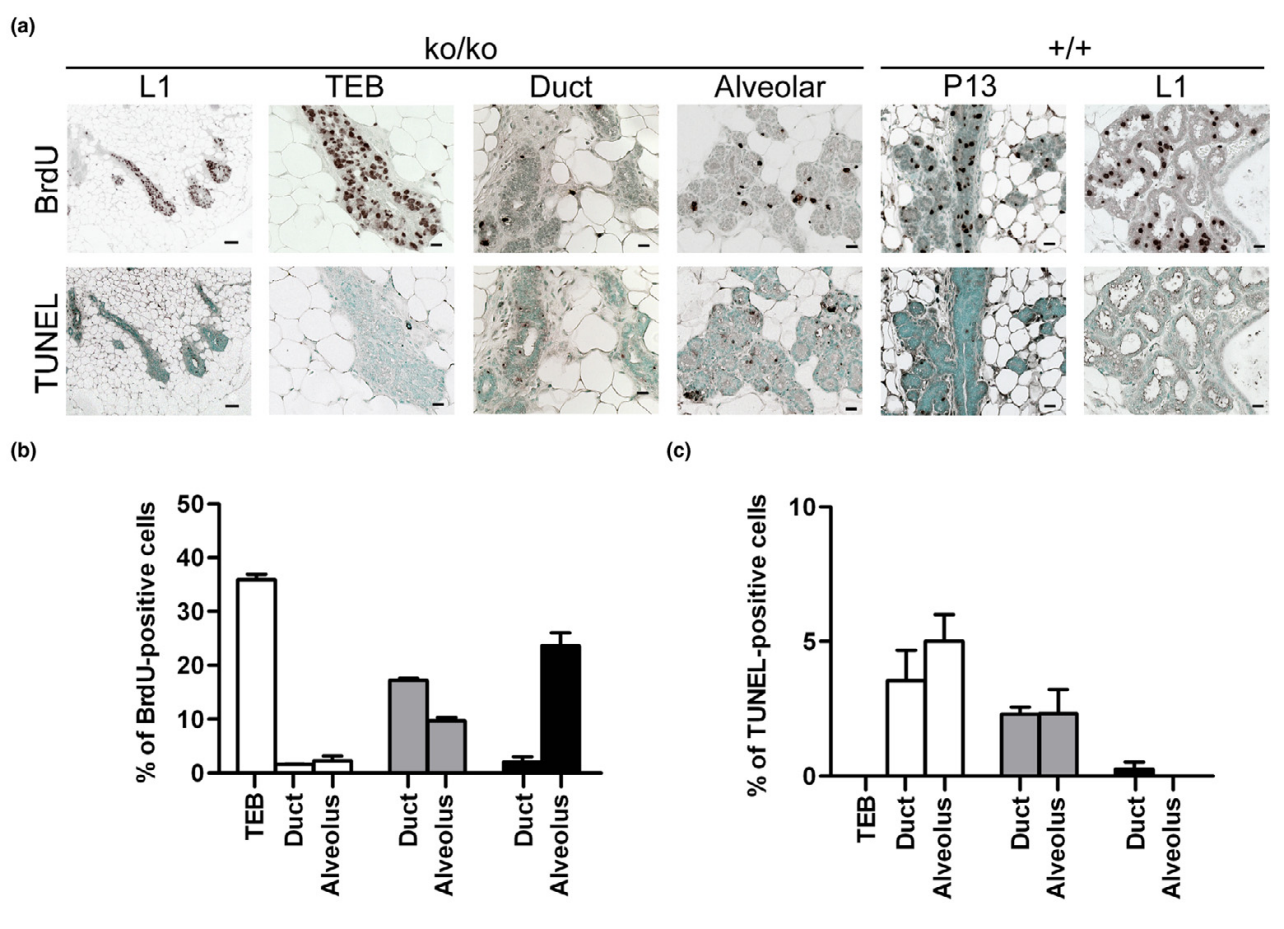
**Mammary epithelial cell proliferation and apoptosis in SirT1^ko/ko ^mice on lactation day 1**. **(a) **Upper panel: bromodeoxyurdine (BrdU) staining of the mammary sections from SirT1^ko/ko ^(ko/ko) and wild-type (+/+) mice on day 13 of pregnancy (P13) and lactation day 1 (L1). From left to right, three terminal end buds (TEBs) at a scale of 100 μm, a single TEB, ducts, and alveolus of SirT1^ko/ko ^mice at a scale of 20 μm, and ducts and alveolus of wild-type mice on P13 and L1 at a scale of 20 μm. Lower panel: TUNEL (Terminal deoxynucleotidyl transferase mediated dUTP Nick End Labeling) assay for apoptotic cells using sections neighbored to the sections for BrdU staining. **(b) **The quantitative analysis of BrdU-positive epithelial cells in TEBs, ducts, and alveoli of SirT1^ko/ko ^mice on L1 (open bar) and wild-type mice on P13 (grey bar) and L1 (solid bar). **(c) **The quantitative analysis of TUNEL-positive cells in neighboring sections.

The finding of pregnancy-induced mammary gland development prompted us to investigate how mammary epithelial progenitor cells in pregnant SirT1^ko/ko ^mice can differentiate so rapidly. Both BrdU labeling and the TUNEL (Terminal deoxynucleotidyl transferase mediated dUTP Nick End Labeling) assay were used to measure the cell proliferation and apoptosis in the mammary tissues, respectively. We found in SirT1^ko/ko ^mammary tissues that about one-third of the cells in TEBs were BrdU-positive as compared with the number of BrdU-positive cells in the newly differentiated ductal or alveolar epithelial cells (Figure 5a(upper panel), 5b). This BrdU-positive finding differs from that in wild-type mammary tissues, in which less than 3% of ductal epithelial cells and about 25% of alveolar epithelial cells were BrdU-positive. The newly generated ductal epithelial cells in SirT1^ko/ko ^mice formed elongated ducts and side branches, while pushing TEBs towards the edge of mammary fat pad (Figure 3a, lower panel). The newly generated ductal epithelial cells can consequently further differentiate into a few observed alveolar cells in which milk proteins were detected (Figure 2c). These observations suggested that the proliferation and differentiation of epithelial progenitor cells, which is programmed to take place at different times of a female's reproductive life, could be concurrent at a late stage of pregnancy in SirT1^ko/ko ^mice.

Apoptosis is a part of the self-renewal and remodeling processes in normal mammary tissues [17,24,25]. Apoptotic epithelial cells can be readily identified in both ductal and alveolar epithelium of SirT1^ko/ko ^mice on lactation day 1 (Figure 5a, lower panel). The levels of apoptotic cells were significantly higher than those in wild-type mice on lactation day 1, but seemed to be closer to that in wild-type mice in mid-pregnancy (e.g. day 13 of pregnancy) (Figure 5c). The rush of proliferation and differentiation during pregnancy-rescued ductal development in SirT1^ko/ko ^mice manifested multiple features on lactation day 1 that could reassemble some characteristic features of wild-type mice in mid-pregnancy. Successive pregnancies, however, failed to improve the survival rate of either SirT1^+/ko ^pups or SirT1^ko/ko ^pups (three or four pregnancies in 3 months for each of three SirT1^ko/ko ^females tested), indicating that the lactation defect persisted. A SirT1 deficiency therefore affected ductal morphogenesis in virgin mice, and lobuloalveolar proliferation in parous mice.

### Deregulated negative feedback signals

The characterization of lactation failure has revealed two developmental defects in SirT1^ko/ko ^female mice. The impeded ductal morphogenesis in virgin SirT1^ko/ko ^mice bore some resemblance to the defective ductal morphogenesis seen in mice lacking GH, estrogen, or IGF-1, as well as in mice harboring mutations in the corresponding receptors, such as the GH receptor and ERα [17,24-31]. These previous studies have established the notion that GH and estrogen synergistically stimulate ductal morphogenesis, which is mediated by stromal cell-produced local IGF-1 in mammary tissues. Impeded ductal morphogenesis in virgin SirT1^ko/ko ^mice is therefore probably due to a deficit in IGF-1 signaling.

In addition to this early defect, pregnancy-induced mammary gland development in SirT1^ko/ko ^mice appeared to be arrested at the beginning of lobuloalveolar development, which is reminiscent of the phenotype in mice lacking IκB kinase alpha (IKKα) activity for NF-κB activation [37]. IKKα-deficient mice exhibit normal ductal morphogenesis at the onset of puberty and exhibit normal ductal side branching during early pregnancy. Owing to the absence of NF-κB-dependent cyclin D_1 _expression, however, IKKα-deficient mice display defective alveolar epithelial cell proliferation and fail to lactate [37]. While SirT1 negatively regulates the transcription activity of FoxOs and NF-κB in mammalian cells [10,11,20], SirT1^ko/ko ^mice displayed the loss-of-function phenotypes. We hypothesized that SirT1 deficiency deregulates the expression of negative feedback signals and thereby desensitizes the cells to various types of stimulation. Indeed, among the many FoxOs and NF-κB-regulated genes, IGFBP-1 and IκBα encode negative feedback signals for IGF-1 signaling and NF-κB activation, respectively. We found that the basal levels of both IGFBP-1 and IκBα were increased in adipose tissues from virgin SirT1^ko/ko ^mice and in ductal epithelial cells from SirT1^ko/ko ^mice on lactation day 1 (Figure 3b). Meanwhile, the expression pattern of IGFBP-1 and IκBα in wild-type mammary tissues, shown in Figure 3b, was in agreement with the findings of others [16,38,39]. Namely, the expression of IGFBP-1 was downregulated during pregnancy, whereas NF-κB activity was increased at a late stage of pregnancy. The loss of SirT1 therefore increased the basal level of both IGFBP-1 and IκBα in multilineages of cells, and potentially increased thresholds for activating SirT1-modulated signaling pathways.

SirT1 deficiency did not affect the ability of mammary epithelial progenitor cells to differentiate into functional alveolar epithelial cells despite the lactation failure (Figure 2c). SirT1 deficiency may therefore alter the homeostasis of the signals for ductal morphogenesis and/or the cellular response to the signals. Increased expression of both IGFBP-1 and IκBα in adipose tissues and mammary epithelial cells could simply reflect a global deregulation of gene expression in SirT1^ko/ko ^cells in which the activity of both FoxOs and NF-κB is increased. IGFBP-1 is a potent inhibitor of IGF-1 *in vivo *[40], suggesting that the elevated basal level of IGFBP-1 in adipose tissues in SirT1^ko/ko ^mice could reduce the effect of IGF-1. Moreover, deregulated IGFBP-1 expression, but not deregulated IκBα expression, was reversed in response to increased levels of estrogen in the mammary fat pads after implantation (Figure 4b). The finding is relevant to the fact that the expression of IGFBP-1 is downregulated as the level of estrogen increases in pregnant wild-type mice (Figure 3b). Increased levels of estrogen therefore stimulate the production of stromal cell-derived local IGF-1, which overcomes the IGFBP-1 barrier in SirT1^ko/ko ^mice and, ultimately, reverses the increased levels of IGFBP-1 in mammary tissues.

**Figure 6:**
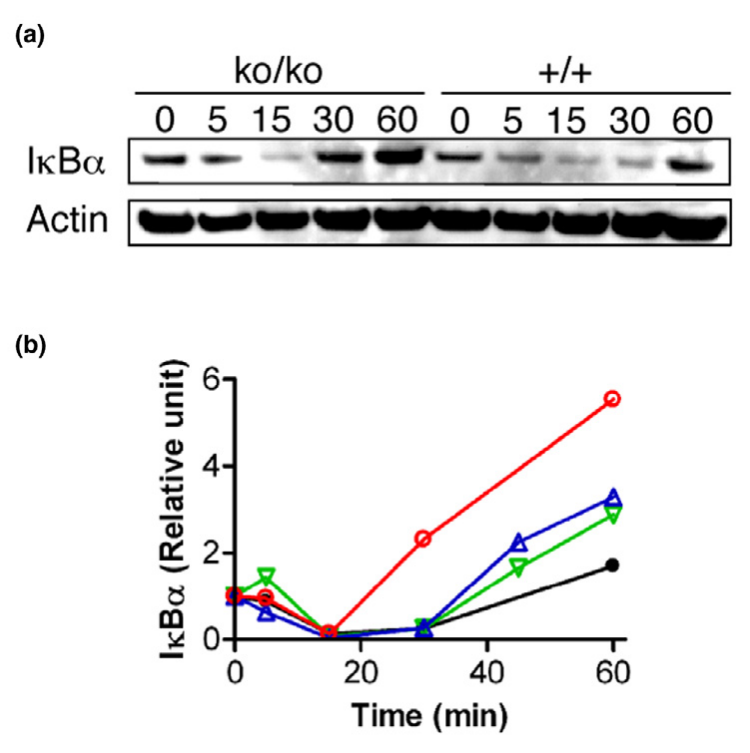
**SirT1 deficiency derails NF-κB signaling in response to TNFα stimulation**. **(a) **Western blot analysis of IκBα expression in SirT1^ko/ko ^(ko/ko) and wild-type (+/+) murine embryonic fibroblasts (MEFs) within the first hour after TNFα stimulation. Actin is used as a loading control. **(b) **The relative levels of newly synthesized IκBα in three independent SirT1^ko/ko ^MEF lines (red circles, blue and green triangles) and wild-type MEFs after TNFα stimulation. For each line of MEFs, the unit of IκBα protein at each time point is relative to the unit 0 minutes after normalization with actin.

It was noted that increased levels of estrogen and local IGF-1 did not interfere with the expression of IκBα (Figure 4b). To determine whether deregulated IκBα expression in SirT1^ko/ko ^cells can be reversed, we treated MEFs with TNFα and measured the kinetics of IκBα expression. Three independent lines of SirT1^ko/ko ^MEFs were used, which displayed varying basal levels of increased IκBα expression. Following IκBα degradation induced by TNFα, the levels of newly synthesized IκBα in all three SirT1^ko/ko ^MEFs were always higher than that of wild-type MEFs (Figure 6). This *in vitro *finding implies that SirT1 deficiency affects NF-κB signaling when the IκB kinase activation is normal.

Our current system might not be suitable for studying the effects of SirT1 deficiency on NF-κB activation *in vivo*. NF-κB signaling exhibits distinct biological responses because the expression of IκBα depends on NF-κB activation while the other isoforms of IκB (β and ε) are independent of NF-κB feedback [41]. For example, TNFα stimulation induces dampened oscillatory behavior after the first hour, whereas lipopolysacharide treatment leads to stable NF-κB activation [42,43]. The NF-κB signaling in the developing mammary gland is under the temporal control of RANK signaling and IKKα activation [37]. The finding of increased levels of newly synthesized IκBα within the first hour indicated that SirT1 deficiency could disrupt the temporal control of NF-κB signaling to all types of stimulation (Figure 6). It remains unknown, however, whether the reduced IGF-1 signaling would affect the efficacy of RANK signaling and IKKα activation in developing mammary glands. The fact that individual SirT1^ko/ko ^mice manifested varying degrees of ductal morphogenesis (Figure 3a) makes it difficult to dissect the potential compound effect of SirT1 deficiency on both upstream IKKα activation and downstream NF-κB signaling. Other experimental systems, such as mammary epithelial cell-specific SirT1^ko/ko ^female mice, may be used to address this issue.

## Discussion

Our study of SirT1^ko/ko ^mice has unveiled a regulatory mechanism by which SirT1 modulates the efficacy of estrogen-stimulated local IGF-1 signaling for ductal morphogenesis. SirT1 deficiency alters the homeostasis of gene expression and attenuates the efficacy of IGF-1 signaling. As a result, SirT1^ko/ko ^mice manifest partial perinatal lethality and lactation failure phenotypes, implicating the role of evolutionarily conserved SirT1 in both postnatal survival and offspring survival of mammals.

### SirT1 balances the homeostasis of IGF-1 and IGFBP-1 *in vivo*

**Figure 7:**
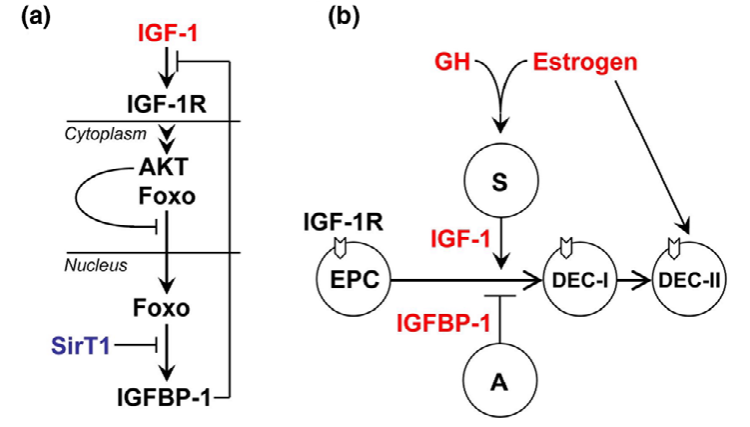
**SirT1 modulates estrogen–insulin-like growth factor-1 signaling for ductal morphogenesis: a model**. **(a) **The insulin/insulin-like growth factor-1 (IGF-1) signaling system: both SirT1 and Akt kinases can negatively regulate the transcription activity of forkhead box 'other' protein (FoxO) proteins. SirT1 deficiency deregulates the expression of insulin-like growth factor-1 binding protein-1 (IGFBP-1), which may exert autocrine and/or paracrine effects to inhibit IGF-1. **(b) **Mammary epithelial precursor cells (EPC) express IGF-1 receptor (IGF-1R) and can differentiate into estrogen receptor (ER)-negative ductal epithelial cells (DEC-I) in response to maternal, circulating, or estrogen-stimulated, stromal cell (S)-derived local IGF-1. At the onset of puberty, ovarian estrogen, in synergy with growth hormone (GH), enhances the production of local IGF-1 and stimulates the differentiation of DEC-I to ER-positive ductal epithelial cells (DEC-II). SirT1 deficiency deregulates the expression of IGFBP-1 in adipose tissues (a), however, which attenuates the efficacy of the IGF-1 signaling and causes impeded ductal morphogenesis in virgin SirT1^ko/ko ^mice. Either pregnancy or exogenous estrogen can overcome the impeded ductal morphogenesis in virgin SirT1^ko/ko ^mice and can stimulate the differentiation of EPC.

SirT1 is an integral part of the IIS system that checks and balances the efficacy of insulin and IGF-1 signals. The IGF-1 signaling of the IIS system is required for survival after birth. As illustrated in Figure 7a, the function of SirT1 parallels the linear IGF-1/IGF-1 receptor/phosphoinositide-3'-OH kinase/Akt signaling pathway, both of which can negatively regulate the transcription activity of FoxOs. Mice harboring targeted mutations in either IGF-1 or the IGF-1 receptor gene exhibit perinatal lethality and growth retardation [44,45]. In the IGF-1 receptor-expressing cells, the binding of insulin and/or IGF-1 activates the phosphoinositide-3'-OH kinase and Akt kinase cascade for survival [8,9,13]. The phosphoinositide-3'-OH kinase/Akt signaling pathway is also highly conserved from worms to mammals, and functions to modulate energy metabolism and lifespan in lower organisms. The mammalian Akt kinase family has three members, Akt1–Akt3, which provide specificity and versatility. Mice lacking both the Akt1 and Akt2 genes display perinatal lethal and growth retardation phenotypes that are strikingly similar to that of either IGF-1 or IGF-1 receptor-deficient mice [46]. In this regard, SirT1^ko/ko ^mice, as well as other strains of SirT1-deficienct mice, also exhibit perinatal lethal and growth retardation phenotypes (Figure 1c and Table 1) [22,23]. The consistent observation of perinatal lethal and growth retardation phenotypes from our study and studies with mice lacking IGF-1, the IGF-1 receptor, or Akt1/Akt2 suggests that upregulated FoxO activity unleashes the expression of downstream effector genes that lead to the birth-related stress. This birth-related stress may be related to the switch from a maternal level of IGF-1 to a neonatal level of IGF-1 because the surviving SirT1^ko/ko ^mice can live into adulthood and can reproduce despite the growth retardation phenotype (Figure 1c). In this context, at least one of the FoxO-deficient mice strains, FoxO3 (Foxo3a/FKHRL1)-deficient mice, grow normally and develop functional mammary glands [47].

IGFBP-1 is one of six IGF-1 binding proteins, but is the only one that can be negatively regulated by insulin [48]. IGF-1 binding proteins inhibit the action of local IGF-1 in a spatial and temporal manner [40,49]. In mammalian cells, FoxO3 can directly bind to the promoter of the IGFBP-1 gene and activate the transcription [50-52]. SirT1 acts to negatively regulate the activity of nuclear FoxO3 and to decrease the expression of IGFBP-1 when the IGF-1 signal is low (Figure 7a). Increased levels of IGFBP-1 can diminish the action of IGF-1 signals *in vivo*. In that way, SirT1 balances the homeostasis of IGF-1 and IGFBP-1 for varying levels of IGF-1 signals.

The homeostasis of IGF-1 and IGFBP-1 clearly affects fertility, as shown by the infertility phenotype in both IGF-1-deficient mice and IGFBP-1 transgenic mice [28,40,49]. The serum level of estrogen in IGF-1-deficient female mice appears significantly reduced, whereas in ERα-deficient mice, which are also infertile, there is a threefold increase [53]. In contrast, SirT1^ko/ko ^mice can be fertile despite the fact that the fertility was reduced as compared with that of wild-type females (Table 1). Although our preliminary assessment indicated that the serum level of estrogen in SirT1^ko/ko ^female mice was not overtly altered, it remains possible that a subtle irregularity or an individual variation of the estrogen level or ERα expression might be attributed to the reduced fertility. An alternative explanation for reduced fertility is that SirT1 deficiency deregulates the activity of FoxO3 and manifests an opposite effect of FoxO3 deficiency. FoxO3-deficient mice exhibit early depletion of ovarian follicles, which is reminiscent of premature ovarian failure in women [47,54]. Premature ovarian failure manifests as early cessation of ovarian function and as premature menopause caused by genetic mutations, chemo/radiation therapy, or other unknown factors. It is possible that SirT1 deficiency counterbalances normal release of ovarian follicles and reduces the fertility in mice.

### SirT1 modulates the efficacy of the IGF-1 signal for ductal morphogenesis

We hypothesize that, in virgin SirT1^ko/ko ^mice, increased levels of IGFBP-1 in adipose tissues effectively diminish IGF-1 signals, including the estrogen–IGF-1 signaling, and halt the differentiation of mammary epithelial progenitor cells (Figure 7b). Several results of this study provided support for this hypothesis. First, the expression of IGFBP-1 is increased in mammary fat pads from virgin SirT1^ko/ko ^mice, in which there is no evidence of any duct (Figures 2a and 3a). Second, both pregnancy and estrogen implantation can rescue impeded ductal morphogenesis in SirT1^ko/ko ^mice (Figures 3a and 4a), demonstrating that increased levels of estrogen can enhance the production of local IGF-1. Moreover, the varying degrees of rescue among individual mice, as well as individual mammary fat pads of the same mouse, also indicated that local IGF-1 must be halted in SirT1^ko/ko ^mice (Figure 3a, and data not shown). Finally, increased levels of estrogen specifically activate the IGF-1 signaling in mammary tissues, as shown by the reversal of IGFBP-1 expression in SirT1^ko/ko ^mice after estrogen implantation (Figure 4b). These results indicate that a gradient of IGF-1 signals, including both circulating and local IGF-1, may regulate mammary gland development at embryonic, postnatal, and reproductive stages, and that increased expression of IGFBP-1 decreases the efficacy of IGF-1 signals.

Mammary development may undergo estrogen/IGF-1-independent phases and estrogen/IGF-1-dependent phases, which encompasses a late stage of embryonic development to the onset of puberty and involves maternal IGF-1, circulating IGF-1, and estrogen-stimulated local IGF-1. By embryonic day 16.5, mammary mesenchymes are connected to primitive fat pads and begin to proliferate, which results in rudimentary ducts [17,25,55,56]. We propose that this process marks the transition from estrogen/IGF-1-independent mammary stem cells to IGF-1-dependent epithelial progenitor cells, and that maternal IGF-1 is sufficient to stimulate the differentiation of epithelial progenitor cells to estrogen-independent ductal epithelial cells but not estrogen-dependent epithelial cells (Figure 7b). This explains why the development of rudimentary ducts is normal in either IGF-1 or ERα knockout mice and that TEBs form and regress immediately before and after birth, respectively [17,26,27]. During the prepubertal period, the rudimentary ducts grow isometrically under the influence of circulating IGF-1. Like maternal IGF-1, circulating IGF-1 is not sufficient to induce the differentiation of estrogen-independent ductal epithelial cells into estrogen-dependent epithelial cells, which will express ERα and become estrogen dependent (Figure 7b). At the onset of puberty, ovarian estrogen, in synergy with pituitary GH, causes a surge in the production of stromal cell-derived local IGF-1 and stimulates the differentiation of estrogen-independent ductal epithelial cells to estrogen-dependent epithelial cells, which begins postnatal development of mammary gland (Figure 7b). Impeded ductal morphogenesis results in virgin SirT1^ko/ko ^mice because local IGF-1 is apparently not sufficient to overcome increased IGFBP-1 in mammary adipose tissues. SirT1 therefore positively regulates the efficacy of the estrogen–IGF-1 signaling for ductal epithelial cell proliferation and differentiation.

### The potential effect of modulating SirT1 activity

Breast cancer results from the accumulation of inherited and/or somatic mutations. Lifetime exposure to estrogen is a major risk factor for breast cancer, and a number of lifestyle factors, such as diet, body fat, alcohol consumption, exercise, parity, and hormone replacement therapy, may influence a woman's exposure to estrogen. The compound personal risk to a woman is difficult to assess because the molecular link between these factors and the effect of estrogen is poorly understood. The result of this study demonstrated that SirT1 positively modulates the efficacy of the estrogen–IGF-1 signal. This suggests that if SirT1 serves as a lifetime sensor to dietary restriction and acute withdrawal of nutrients [14,57], its activity could ultimately influence the risk of breast cancer. Interestingly, an epidemiological study of Dutch famine in 1945 indicated that an exposure to famine at prepubertal age increased the risk of breast cancer, most clearly seen in women who never gave birth [58]. Moreover, the serum levels of estrogen and IGF-1 were increased among those exposed to the famine, which suggests that an epigenetic adaptation phenomenon may take place. Assessing the potential correlation between the enzymatic activity of SirT1 and the risk of breast cancer is therefore of great interest in order to identify feasible targets for chemoprevention against breast cancer.

Several dietary polyphenols as well as small molecules have been identified as either agonists or antagonists of SirT1 [59,60]. The pharmacological effects of these compounds in humans remain elusive given that SirT1 targets multiple transcription factors and exerts pleiotropic effects. Nonetheless, our study of SirT1^ko/ko ^mice demonstrated the potential utility of SirT1 antagonists. Specifically, reducing SirT1 activity alters the homeostasis of cellular responses, including deregulation of the expression of IGFBP-1 and IκBα, which can attenuate the stimulation from the estrogen–IGF-1 signaling and potentially desensitize mammary epithelial cells to NF-κB activation. SirT1 is therefore a candidate target for chemoprevention against breast cancer.

## Conclusion

The study of mammary gland development is an excellent model system for unraveling how SirT1 modulates the efficacy of the estrogen–IGF-1 signaling and regulates the timing of ductal morphogenesis. These new mechanistic insights may also aid in understanding the role of SirT1 in breast cancer as well as in other organs in which local IGF-1 plays an important role.

## Abbreviations

Atm = ataxia telangiectasia; BrdU = bromodeoxyurdine; CMV = cytomegalovirus; ERα = estrogen receptor alpha; FoxO = forkhead box 'other' protein; GH = growth hormone; IGF-1 = insulin-like growth factor-1; IGFBP-1 = insulin-like growth factor-1 binding protein-1; H & E = hemotoxylin and eosin; IIS = insulin/insulin-like growth factor-1 signaling; IκBα = inhibitors of NF-κB alpha subunit; IKKα = IκB kinase alpha; MEF = murine embryonic fibroblast; NF = nuclear factor; PCR = polymerase chain reaction; RANK = receptor activator of NF-κB; Sir = silencing information regulator; TEB = terminal end bud; TNF = tumor necrosis factor.

## Competing interests

The authors declare that they have no competing interests.

## Authors' contributions

HL characterized the phenotypes of the SirT1 mutant mice, participated in the design of the study, and helped to draft the manuscript. GKR and NL helped to characterize the phenotypes. CW generated the SirT1 mutant embryonic stem cells and mice. BPR helped analyze the histology data. YG conceived of the study, participated in its design and coordination, and drafted the manuscript. All authors read and approved the final manuscript.

## Supplementary Material

Additional file 1 A pdf file containing the materials and methods for the generation of SirT1^ko/ko ^mice. This supporting file documented the details of materials and methods used to generate both SirT1^co/co ^mice and SirT1^ko/ko ^mice. Click here for file

## Figures and Tables

**Table 1:** Perinatal lethality, fertility, and lactation defect in SirT1^ko/ko ^mice

Parents		Pups				
Male	Female	Litter	Survivors^a^	+/+	+/ko	ko/ko

+/ko	+/ko	47	340	101	208	31
ko/ko (*n *= 3)	+/ko	11	55	-	45	10
+/ko	ko/ko (*n *= 5)	8	0	-	(13)	(13)

More than 10 pairs of SirT1^+/ko ^male mice and SirT1^+/ko ^female mice were used for breeding. Data in parentheses indicate the number of pups of this genotype found dead within 3 days after their birth. ^a^Total number of surviving pups at weaning (postnatal day 21).
