# Supplementary material for: SirT1 modulates the estrogen–insulin-like growth factor-1 signaling for postnatal development of mammary gland in mice
Source: Breast Cancer Res. 2007 Jan 3;9(1):R1. doi: 10.1186/bcr1632 (PMC1851382; doi:10.1186/bcr1632)
Supplement: Additional file 1 — A pdf file containing the materials and methods for the generation of SirT1ko/ko mice. This supporting file documented the details of materials and methods used to generate both SirT1co/co mice and SirT1ko/ko mice. [file bcr1632-S1.pdf]

SUPPORTING ONLINE MATERIALS

1. Generation of SirT1$^{co/co}$ mice.

The SirT1 gene-targeting vector, KOII [22], was constructed by YG while in the laboratory of Dr. Alt at Harvard Medical School, Boston. Linear KOII DNA was used to transfect R1 ES cells. Both 5' and 3' probes were used in the southern blot analysis for identification of ES clones harboring a correctly targeted locus, designated as SirT1$^{+/co}$ (figure 1a). SirT1$^{+/co}$ ES cells were injected into blastocysts isolated from C57B6 female donor mice. The donor females were super-ovulated three days early by using hCG (at 5IU per mouse injection, 48 hours after PMS injection) and then mated with the males. Plugged females were separated from the male mice, and were sacrificed three days later. The blastocysts were flushed out from the uteri for ES cell injection. The resultant blastocysts were implanted by uterine transfer into a foster mother that was pseudo-pregnant by a vasectomized male (purchased from Charles River). The foster mother was anesthetized. A small incision was made laterally to expose the abdominal cavity. The end of the fundibulum was gently pulled out by holding the fat pad and the blastocysts were expelled into the uterine cavity. The mouse was closed with wound clips and allowed to recover. Clips were removed 7-10 days post surgery. After birth, the pups displaying a high percentage of chimerism of injected R1 ES cells were chosen for back crossing with wild type C57B6 mice. The germline-transmitted SirT1$^{+/co}$ mice can be identified based on both the agouti coat color and genotype of tail DNA. SirT1$^{+/co}$ mice were interbred to generate SirT1$^{co/co}$ mice. Both SirT1$^{+/co}$ and SirT1$^{co/co}$ were phenotypically normal and thus SirT1$^{co/co}$ mice were used for the maintenance of the colony. This inbred SirT1$^{co/co}$ colony is in a mixed 129SvJ/C57B6 background.

2. Generation and characterization of SirT1$^{ko/ko}$ embryonic stem cells

To validate whether SirT1$^{+/co}$ locus can readily be converted to SirT1$^{+/ko}$ locus and to obtain SirT1$^{ko/ko}$ ES cells, the SirT1$^{+/co}$ ES clones were cultured in ES medium in the presence of high concentrations of G418 and the surviving colonies were genotyped for identification of SirT1$^{co/co}$ ES colonies. The SirT1$^{co/co}$ ES cells were transfected with a CMV-Cre expression construct. The Cre recombinase, encoded by the CMV-Cre construct, mediates DNA recombination between two lox sites in the targeted loci in the cells. Several forms of deletion configurations were expected. However, SirT1$^{ko/ko}$ ES clones can be readily identified when both copies of neomycin-resistance gene along with exon 4 of the SirT1 gene were deleted (figure S1a and S1b). In addition, SirT1$^{ko/ko}$ ES clones became sensitive to G418 treatment in contrast to their parental cells. RT-PCR analysis demonstrated that SirT1$^{ko/ko}$ ES cells express a transcript short of the sequences corresponding to exon 4 (fig. S1c).

3. Generation and characterization of SirT1$^{ko/ko}$ mice

To generate SirT1$^{ko/ko}$ mice, SirT1$^{co/co}$ mice were bred with CMV-Cre transgenic mice, which was in C57B6 background. A PCR-based genotyping method, described in Materials and Methods, was used to identify SirT1$^{+/ko}$ mice among the offspring of SirT1$^{+/co}$ mice as shown in figure S1d. To validate whether these SirT1$^{+/ko}$ mice carried a germline-transmitted mutation, the mice were bred with wild type mice and their SirT1$^{+/ko}$ offspring were used to establish a SirT1$^{ko/ko}$ breeding colony (fig. S1e).

To isolate MEFs, SirT1$^{+/ko}$ females were mated with SirT1$^{+/ko}$ males and their embryos were isolated between day 12.5 and day 14.5 and fibroblasts were purified in DMEM-based MEF culture medium.

Li et al., figure S1

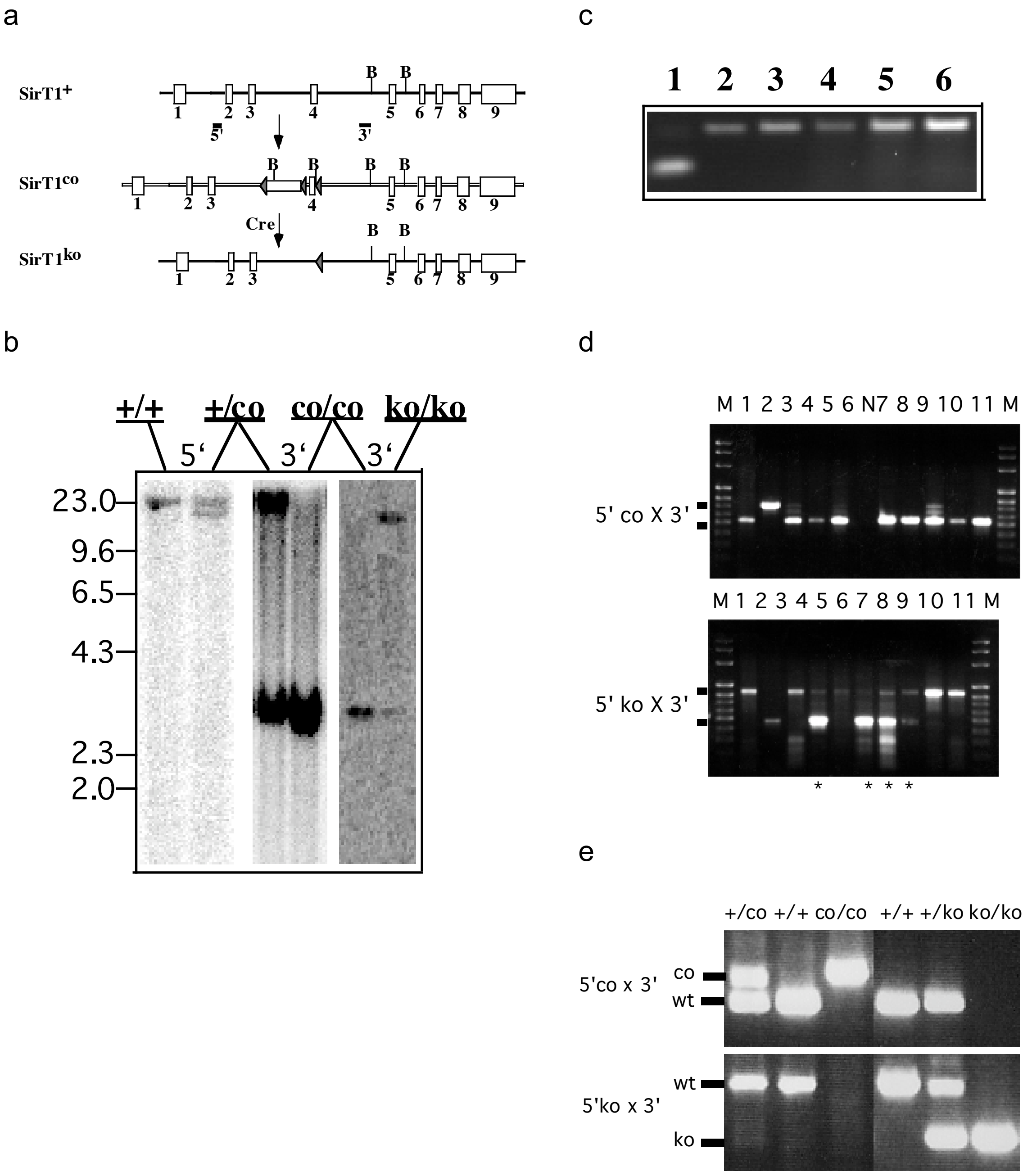

figure S1. Generation of SirT1$^{co/co}$ mice, SirT1$^{ko/ko}$ ES cells, and SirT1$^{ko/ko}$ mice. **a.** Configuration of wild type (SirT1^{+}), conditional targeted (SirT1^{co}), and SirT1 knockout (SirT1^{ko}) loci in the SirT1 gene. "B" denotes BamHI. **b.** Southern blot analysis of ES cells of various genotypes identified by using either 5' or 3' probe on BamHI-digested genomic DNA. **c.** RT-PCR analysis of purified ES colonies of different genotypes. 1, SirT1$^{ko/ko}$; 2, SirT1$^{co/co}$ after CMV-Cre transfection; 3, SirT1$^{co/co}$ before transfection; 4, SirT1$^{+/co}$ after G418 selection; 5, parental SirT1$^{+/co}$ for G418 selection; 6, wild type parental ES cells for KOII SirT1 gene targeting. **d.** Genotypes of a F1 offspring (line 3 to 11) from breeding of a SirT1$^{co/co}$ female to a male CMV-Cre transgenic mouse. The upper panel shows the genotypes for SirT1 co locus, whereas the lower panel shows the genotypes for SirT1 ko locus. Line 1, wild type TC1 ES cells; line 2, the upper panel is SirT1$^{co/co}$ ES cells and the lower panel is SirT1$^{ko/ko}$ ES cells. * marks the mouse for testing germline transmission of its ko allele. **e.** Samples of typical results for cells, tissues, and mice of all genotypes.
